# Supplementary material for: Multi-Method Quantification of Acetyl-Coenzyme A and Further Acyl-Coenzyme A Species in Normal and Ischemic Rat Liver
Source: Int J Mol Sci. 2023 Oct 6;24(19):14957. doi: 10.3390/ijms241914957 (PMC10573920; doi:10.3390/ijms241914957)
Supplement: Supplementary file 1 [file ijms-24-14957-s001.zip › ijms-2624428-supplementary.pdf]

International Journal of *Molecular Sciences*

MDPI

SUPPLEMENTARY MATERIAL

Malgorzata Tokarska-Schlattner, Nour Zeaiter et al. (2023): Multi-method quantification of acetyl-CoA and further acyl-CoA species in normal and ischemic rat liver.

Table S1. Acyl-CoA standards determined by LC/MS-MS

Acyl-CoA species		m/z	Retention time (min)	Concentration/signal [3]		Limit of detection [4]	
Name [1]	Number of acyl			Linear range (nM)	R	Quantity (fmol)	Concentration (nM)
CoA	/	768,1225	2,82	4 - 3000	0,98	< 0,5	< 0,05
acetyl-CoA	C2	810,1331	2,82	4 - 3000	0,99	5	0,5
propionyl-CoA	C3	824,1487	2,83	10 - 3000	0,99	100	10
malonyl-CoA	C3O2	854,1230	2,00	10 - 3000	0,99	5	0,5
butyryl-CoA / isobutyryl-CoA [2]	C4	838,1644	6,41	10 - 3000	0,99	< 100	< 10
crotonyl-CoA	C4Δ2	836,1487	2,83	10 - 3000	0,99	< 100	< 10
succinyl-CoA / methylmalonyl-CoA [2]	C4O2	868,1385	2,83	10 - 3000	0,9	40	4
isovaleryl-CoA	C5	852,1806	10,56	10 - 3000	1,00	40	4
methylcrotonyl-CoA	C5O2	850,1644	9,52	10 - 3000	0,99	< 100	< 10
glutaryl-CoA	C5O2	882,1542	2,83	4 - 3000	0,97	40	4
3-hydroxy-3-methyl-glutaryl-CoA	C6O3	912,1647	2,82	10 - 3000	0,96	40	4
octanoyl-CoA	C8	894,2269	15,70	10 - 3000	0,99	<100	<10
lauroyl-CoA	C12	950,2896	15,80	40 - 3000	0,99	<100	<10
palmitoyl-CoA	C16	1006,3522	16,00	ND	ND	ND	ND
oleoyl-CoA	C18Δ9	1032,3678	16,60	ND	ND	ND	ND

1) Color code for CoA esters: blue, hydrophobic; red, acidic. 2) Species not distinguished by MS. 3) Most linear range and 4) lowest quantity or concentration detectable, derived from measured standard mixtures. ND, not detectable.

Table S2. Acyl-CoAs in mild liver ischemia by LC-MS/MS

Acyl-CoA species [1]	Relative abundance [3]	
	Control	Ischemia
acetyl-CoA	1,00 ± 0,22	1,27 ± 0,38
propionyl-CoA	0,05 ± 0,01	0,24 ± 0,10
malonyl-CoA	0,054 ± 0,002	0,07 ± 0,02
butyryl-CoA / isobutyryl-CoA [2]	0,23 ± 0,06	0,66 ± 0,06
crotonyl-CoA	0,0007 ± 0,0004	0,00 ± 0,00
succinyl-CoA / methylmalonyl-CoA [2]	0,29 ± 0,02	0,0032 ± 0,0006
methylcrotonyl-CoA	0,006 ± 0,005	0,11 ± 0,04
glutaryl-CoA	0,010 ± 0,006	0,008 ± 0,003
3-hydroxy-3-methyl-glutaryl-CoA	0,31 ± 0,08	0,09 ± 0,02
lauroyl-CoA	0,16 ± 0,08	0,35 ± 0,15

1) blue: hydrophobic, red: acidic. 2) species not distinguished by MS.
3) Data are mean ± SEM (n=3, 3 technical replicates each).

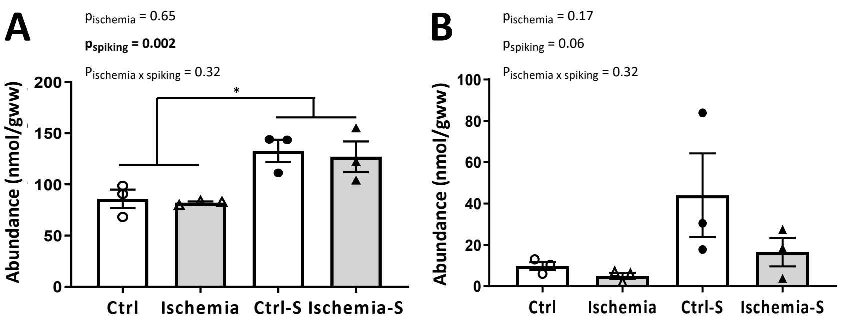

Figure S1. Recovery of internal acetyl-CoA standards in photo- and fluorimetric assays. (A) Spectrophotometric and (B) fluorimetric assays for acetyl-CoA detected in PCA extracts of control liver (white bars) or liver subjected to mild ischemia (grey bars) without and with spiking (55 nmol/g) at the moment of extraction. Data are presented as mean +/- SEM (n=3 animals) and analyzed by two-way Anova; *p < 0,005. Ctrl, control liver, Ischemia, mild ischemic liver, Ctrl-S and ischemia-S, spiked samples.

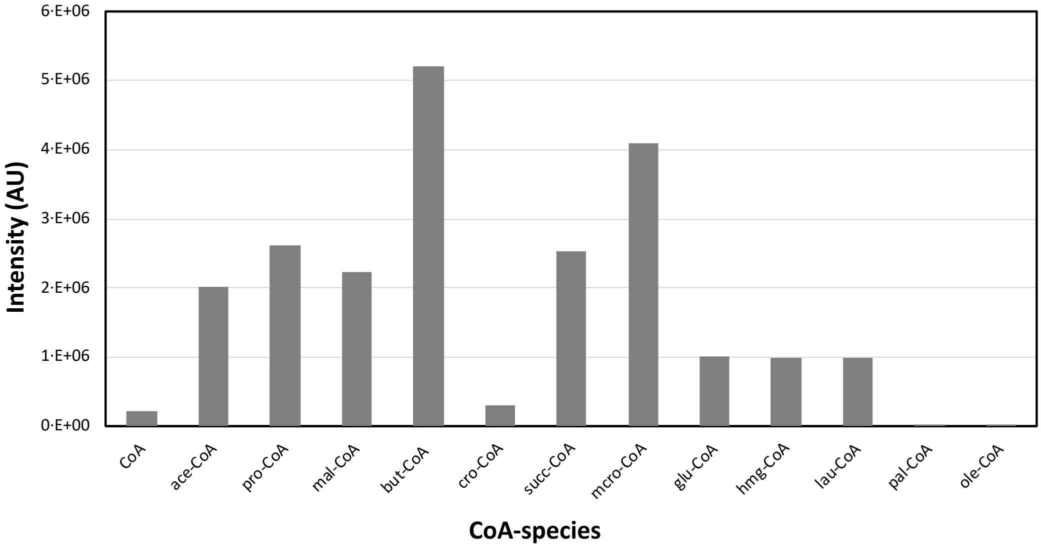

Figure S2. Absolute signal intensity of acyl-CoA standards in LC-MS/MS. The 16 CoA/acyl-CoA standards (see list in Table S1) are compared for their MS signal intensity at an amount of 1,1 pmol. Abbreviations: *ace*, acetyl (C2); *pro*; propionyl (C3); *mal*, malonyl (C3); *but*, isobutyryl and butyryl (C4); *cro*, crotonyl (C4); *succ*, succinyl and methylmalonyl (C4); *isoval*, isovaleryl (C5); *mcro*, methyl-crotonyl (C5); *glu*, glutaryl (C5); *hmglu*, 3-hydroxy-3-methyl-glutaryl (C6); *oct*, octanoyl (C8); *lau*, lauroyl (C12); *pal*, palmitoyl (C16); *ole*, oleoyl (C18). Note: CoA,, palmitoyl-CoA and oleoyl-CoA were excluded from further analysis, based on their low or absent signal with our MS protocol.

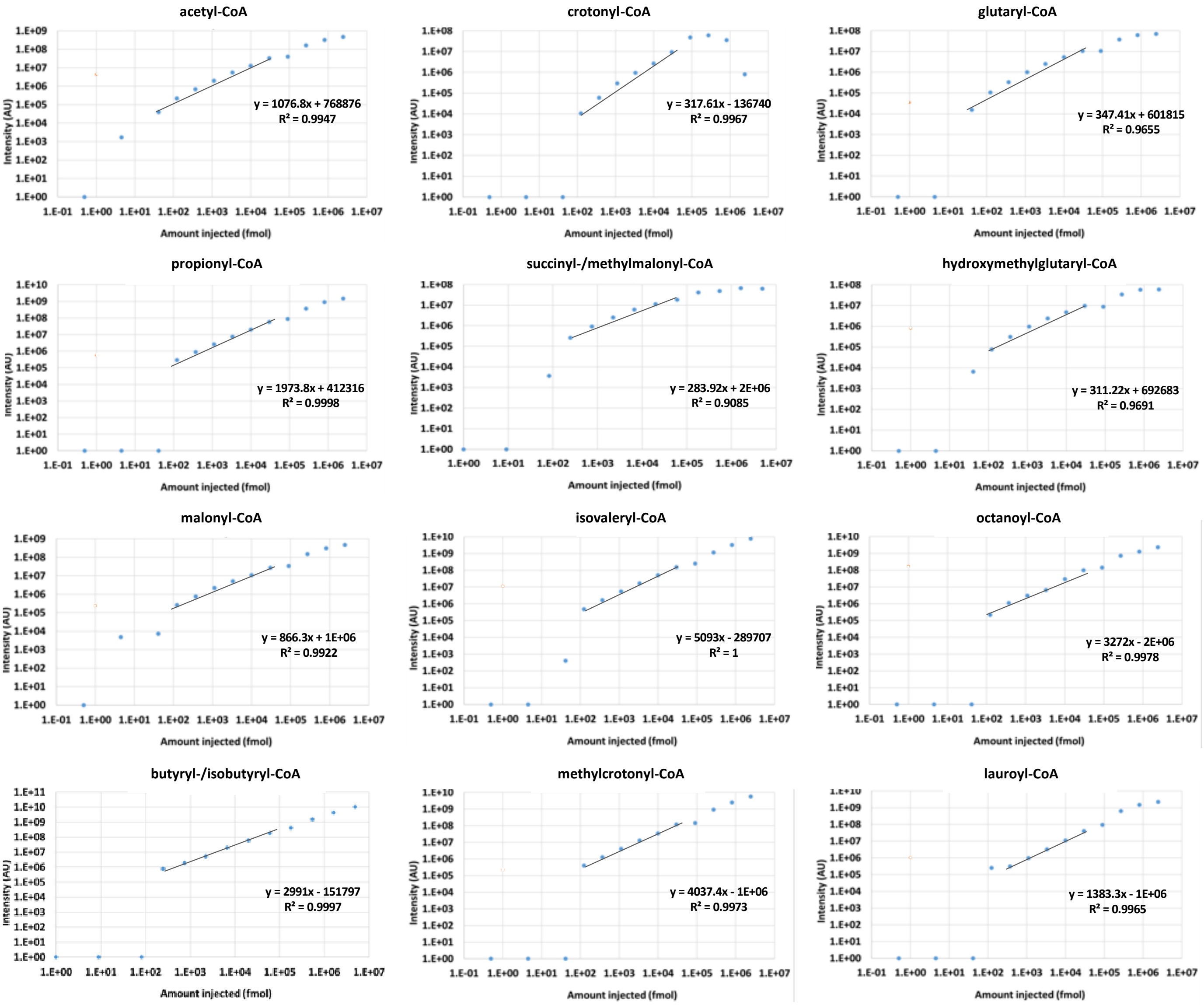

Figure S3. Absolute intensity of acyl-CoA concentration series in LC-MS/MS. Data for CoA species are shown in double logarithmic scale. The regression line drawn in the most linear range (as indicated in Table 1) is indicated.

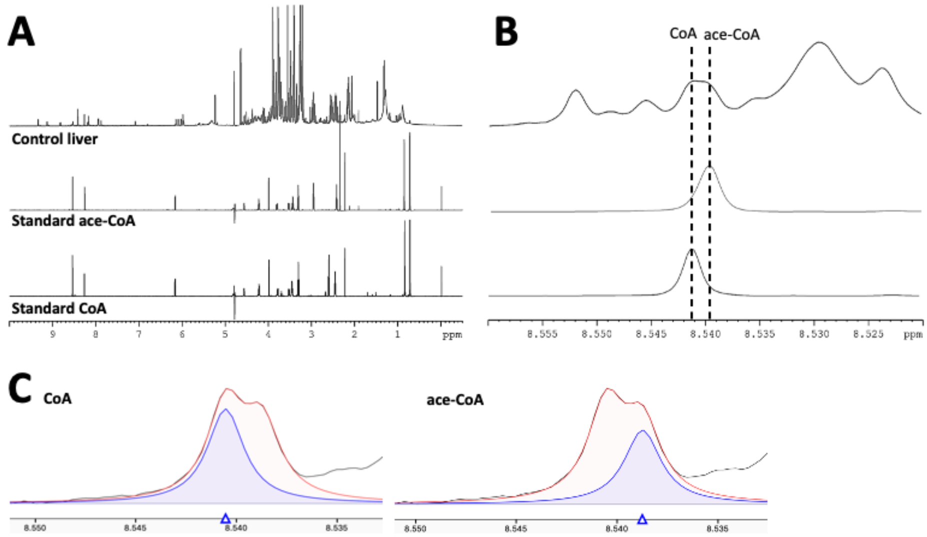

Figure S4. NMR spectra of liver extracts and standards (CoA, acetyl-CoA). (A) Full spectra. (B) Peaks of CoA and acetyl-CoA around 8.54 ppm. (C) Quantification of peaks in (B); red, NMR signal; blue, identified peaks after deconvolution.
